# The Impact of COVID-19 on the Revenue of the Livestock Industry: A Case Study of China

**DOI:** 10.3390/ani11123586

**Published:** 2021-12-17

**Authors:** Jianxiong Chen, Chung-Cheng Yang

**Affiliations:** Department of Accounting, National Yunlin University of Science and Technology, Yunlin 64002, Taiwan; D10825001@gemail.yuntech.edu.tw

**Keywords:** livestock industry, revenue, COVID-19, financial statement perspective, coronavirus

## Abstract

**Simple Summary:**

The purpose of this study is to investigate the impact of COVID-19 on the revenue of the livestock industry and to study the challenges COVID-19 brings to the livestock industry. From the perspective of financial statements, we use the revenue function of listed Chinese livestock companies from 2015 to 2020 to quantitatively estimate the impact of the pandemic. The study results show that the COVID-19 pandemic has reduced the revenue of the livestock industry and has different implications on livestock enterprises of various sizes. Based on the above study results, we provide further suggestions for measures that governments and livestock companies can consider to reduce the impact of the pandemic, thereby increasing the level of sales of livestock products.

**Abstract:**

The COVID-19 pandemic has affected social order and people’s health and has also caused a heavy blow to the livestock industry, affecting animal management and welfare. The livestock industry is one of the main contributors to economic growth in many regions, and it is of great significance to people’s lives and regional economic growth. COVID-19 has reduced the livestock industry’s market as well as consumers’ opportunities to purchase livestock products, resulting in no sales or low sales of livestock or their products. The main purpose of this study is to consider the impact of the pandemic on the revenue of the livestock industry, and to study the challenges arising from the pandemic to the livestock industry. Based on the perspective of financial statements, we estimate the impact of COVID-19 through the translog revenue function of listed Chinese livestock companies from 2015 to 2020, and the study results show that the COVID-19 pandemic has reduced the revenue of the livestock industry, but the decline in revenue of large livestock enterprises is lower than that of small and medium-sized livestock enterprises. In the last two parts of this study, we make policy recommendations to livestock enterprises and the authorities.

## 1. Introduction

How does the livestock industry affect the environment and society? On the one hand, the impact of animal husbandry on climate change is very significant [1,2]. The greenhouse gases produced by livestock account for more than 14.5% of the total global greenhouse gas emissions, exceeding all vehicles, trucks, trains, airplanes, and ships on the planet. The sum of emissions. In the past 40 years, more than 40% of the forests in Central America have been cut down, and 720,000 square kilometers of rainforest in South America have been cut down, all of which provide land for animal husbandry production and growing fodder (mainly soybeans). In addition, animal husbandry occupies one-third of the earth’s freshwater resources, 45% of the earth’s land resources, and one-third of the land is desertified because of animal husbandry. On the other hand, the livestock industry affects people’s diet and health [3], is the foundation of human well-being [4], and plays a vital role in the food supply, culture, and economy in rural and urban areas [5].

Before the COVID-19 pandemic, African swine fever had a significant impact on China’s livestock industry, and more precisely, it had a substantial impact on China’s pig farming. Although African swine fever has brought panic, it has also brought about changes in China’s pig farming. Affected by African swine fever, China’s large-scale pig farming companies have expanded rapidly, and the concentration of the pig farming industry has increased. According to a report from the Ministry of Agriculture and Rural Affairs of China, most African swine fever outbreaks in China before 2020 occurred in small- and medium-sized pig farms. Due to small- and medium-sized pig farms’ low epidemic prevention and risk-bearing capabilities, they are increasingly withdrawing from the market under the pressure of the epidemic and government policy. However, large-scale pig farming companies can increase the rapid detection of African swine fever from aspects of pig farm management and equipment utilization and improve safety guarantees. In the market and government policies, large pig farming companies have used their capital to acquire or build new pig farms to accelerate their expansion. The industry concentration of China’s pig farming industry has rapidly increased. In June 2019, the Ministry of Agriculture and Rural Affairs of China and the Ministry of Finance of China jointly issued the “Notice on Doing a Good Job in Subsidy for Working Capital Loans on Pig Breeding Farms and Large-scale Pig Farms.” This policy provides support for short-term loan discounts to large pig farms. The Chinese government vigorously supports the development of large-scale pig-farming enterprises in terms of policies. In addition, to reduce the risk of African swine fever, downstream slaughter companies also prioritize purchasing slaughter pigs from large pig farms, and large-scale pig farms have become the industry’s mainstream. In addition, African swine fever has also accelerated the integration of the upstream and downstream of China’s pig farming industry chain.

In China, the growth in livestock before the pandemic was mainly the result of increased consumption of pork, beef, and chicken [6,7,8]. From 2000 to 2018, China’s pork production increased from 39.66 million tons to 54.04 million tons, and the proportion of pork in total meat production fell from 65.90% to 62.70%. In 2019, due to the impact of the African swine fever epidemic, the scale of pig farming in China dropped sharply, and the proportion of pork in 2019 dropped sharply to 55.6%. Since the beginning of the new century, China’s total output of cattle, sheep, and poultry meat has increased. From 2000 to 2019, China’s poultry meat production increased from 12.71 million tons to 22.39 million tons, and its proportion rose from 21.1% to 29.3%. During the same period, China’s beef production rose from 5.13 million tons to 6.87 million tons, and its proportion increased from 8.5% to 8.7%. China’s mutton production rose from 2.64 million tons to 4.88 million tons, and its proportion increased from 4.4% to 6.4%. The COVID-19 outbreak has had a significant impact on many sectors at the global, regional, and national levels, including the livestock sector [9,10,11]. Furthermore, the meat, poultry, and animal product processing plants have been the sectors most affected by the COVID-19 pandemic [12,13,14].

Since the discovery of African swine fever in China in 2018, panic has spread throughout the country, which has caused a large number of pig deaths, a shortage of pork products, and a sharp increase in the prices of pork and other meat products [15]. In the COVID-19 pandemic, many countries have adopted measures such as lockdowns and traffic restrictions. The Chinese government has also taken a series of practical actions. For example, to prevent the spread of the virus, farmers’ markets and feed mills were closed [16], slaughterhouses were delayed to resume work, and transportation was restricted [17]. These factors led to a shortage of feed supplies, unsalable livestock products, and increased production and transportation costs [18]. During the pandemic, China implemented strict traffic controls to control the movement of people and vehicles [17], which had a severe impact on the supply of livestock production materials and the sale of products [15]. It is difficult for livestock enterprises and farmers to deliver livestock products to the market [19], and it even leads to the interruption of the entire livestock industry supply chain [15].

Many government relief policies are not enough to make up for the loss of livestock product sales caused by the COVID-19 pandemic [20]. Understanding the specific impact of the pandemic on livestock product sales and the revenue of livestock enterprises, and how to minimize the impact has become a top priority. At present, the quantitative research on the impact of the COVID-19 pandemic on the sales of livestock products and the revenue of livestock enterprises is relatively limited. Based on the perspective of financial statements, we use the revenue function of listed Chinese livestock companies from 2015 to 2020 to quantitatively estimate the impact of the pandemic. The research results show that the COVID-19 pandemic has reduced the revenue of the livestock industry, but the decline in revenue of large livestock enterprises is lower than that of small and medium-sized livestock enterprises. Based on the above research results, we provide further suggestions for measures that governments and livestock companies can consider to reduce the impact of the pandemic, thereby increasing the level of sales of livestock products. The remaining part is as follows: Section 2 provides background and hypothesis development. In Section 3, we introduce the research methods and models. Section 4 and Section 5 are the results and conclusions, respectively.

## 2. Background and Hypothesis Development

When an infectious disease breaks out, the hungry population will increase [21,22], and workers will decrease accordingly. Since the virus can spread from person to person through the air [23], and workers in meat and poultry processing plants need to work closely with each other, this increases the risk of virus transmission [11,23,24,25]. COVID-19 has caused meat and poultry processing plant employees to become sick, putting livestock companies at risk of closing down, which reduces the supply of livestock products [20]. As processing plants are blocked [26,27,28,29,30,31], many poultry producers have had to discard animals because they cannot supply them to meat processing plants [31]. In addition, restrictions on social distancing and transportation have caused sales difficulties, and many dairy farmers have had to dump milk [26]. The closure of restaurants, hotels, and schools has also affected the sales of livestock products [32]. Due to sales difficulties and the high cost of raising animals, this has led to the unfortunate problem of euthanasia of animals [26,33,34].

Due to the COVID-19 pandemic, the labor force of the livestock industry shrank [23,35,36], and many processing plants closed [27,28,29,30,31] or reduced processing capacity [23,30]; livestock companies faced difficulties in slaughtering and processing livestock [20]. The increase in consumption of livestock products has always been a driving force for the development of the livestock industry. However, the COVID-19 outbreak has caused significant losses to all economic sectors including the livestock industry [37], and livestock companies and farmers have been losing their regular customers [38,39,40,41]. The blockade of hotels, schools, and restaurants (the main consumers of animal products) and the tourism slump have led to a sharp decline in market demand for animal products, which further makes it difficult for livestock companies to sell their products [37].

After the outbreak of COVID-19, the sales of animal products on online sales platforms in China showed a significant increase [9], but the sales in the physical market have dropped significantly. In addition, there were rumors that livestock can spread COVID-19 and people should stop buying and eating animal products [42,43,44]. These misunderstandings that led to the belief that livestock or animal products are the hosts or carriers of the virus have left people with the impression that humans may be infected with COVID-19 through the consumption of animal products, which has further exacerbated the decline in sales of meat and other animal products [9]. The interruption of international trade routes also limits the development of livestock enterprises [9,45,46], which may eventually affect the sales of meat products and dairy products export suppliers, and further reduce the income of livestock companies and farmers [9].

The closure of live animal markets in many countries has caused livestock producers to face difficulties in selling animal products, which has greatly reduced the opportunities for livestock products to enter the market for consumers to purchase. In addition, due to traffic restrictions and the fear of being infected by the virus, people have drastically reduced the frequency of going to supermarkets and markets to purchase animal products from on-site stores, which has further led to a decline in sales of animal products. However, even if people reduce the overall frequency of going to the market, in a limited time, Chinese people still tend to buy livestock products produced by large livestock companies. Therefore, we propose the following hypothesis:

**Hypothesis** **1** **(H1).** *The COVID-19 pandemic has reduced the revenue of the livestock industry, but the decline in revenue of large livestock enterprises is lower than that of small- and medium-sized livestock enterprises.*

## 3. Method

### 3.1. Theoretical Model

High-quality employees are one of the prerequisites for the stable operation of enterprises [47,48,49,50,51,52]. We use the following equation to represent the livestock industry production function (The definitions of $x_{1},x_{2},x_{3},f,\;d$ and b are shown in Table 1):

(1) $$y=f\left(x_{1},x_{2},x_{3},f,\;d,\;b\right)$$

In Equation (1), y is the sales, the revenue function of livestock enterprises is as follows:

(2) $$\begin{array}{c} r\left(p;x_{1},x_{2},x_{3},f,\;d,\;b\right)=max\;py\\ subject\;to\;y=f\left(x_{1},x_{2},x_{3},f,\;d,\;b\right)\; \end{array}$$

r is revenue, p represents the prices of livestock products. The revenue function of livestock enterprises is converted into the following equation:

(3) $$lnr=\alpha_{0}+\delta lnp+\overset{3}{\underset{i=1}{\sum }}\alpha_{i}lnx_{i}+\beta_{1}lnf+\delta_{1}ln\;d+\epsilon_{1}ln\;b$$

This study normalized by setting p *=* 1 [53]. we can further simplify Equation (3) as follows:

(4) $$lnr=\alpha_{0}+\overset{3}{\underset{i=1}{\sum }}\alpha_{i}\;ln\;x_{i}+\beta_{1}ln\;f+\delta_{1}ln\;d+\epsilon_{1}ln\;b$$

Scholars have studied the use of translog revenue function in many industries [54]. The translog revenue function in this study is as follows:

(5) $$\begin{array}{cc} ln\;r=\alpha_{0}+\overset{3}{\underset{i=1}{\sum }} & \alpha_{i}\;ln\;x_{i}+\beta_{1}ln\;f+\delta_{1}ln\;d+\epsilon_{1}ln\;b+\frac{1}{2}\overset{3}{\underset{i=1}{\sum }}\overset{3}{\underset{i=1}{\sum }}\alpha_{il}\;ln\;x_{i}\;ln\;x_{l}+\frac{1}{2}\beta_{11}{\left(lnf\right)}^{2}+\frac{1}{2}\delta_{11}{\left(lnd\right)}^{2}\\ & +\frac{1}{2}\epsilon_{11}{\left(lnb\right)}^{2}+\overset{3}{\underset{i=1}{\sum }}\gamma_{i1}lnx_{i}ln\;f+\overset{3}{\underset{i=1}{\sum }}\varepsilon_{i1}lnx_{i}ln\;d+\overset{3}{\underset{i=1}{\sum }}\mu_{i1}lnx_{i}ln\;b+\theta_{11}ln\;f\;ln\;d\\ & +\rho_{11}ln\;f\;ln\;b+\sigma_{11}ln\;d\;ln\;b+\rho_{11}ln\;f\;ln\;b+\sigma_{11}ln\;d\;ln\;b \end{array}$$

### 3.2. Data and Variables

#### 3.2.1. Data Source and Sample Period

We obtained the data for this study from the quarterly financial statements data provided by the CSMAR database, including the livestock industry data from 2015 to 2020. We have excluded unreasonable observations, such as the number of employees or zero net fixed assets. Finally, the livestock industry obtained 255 valid observations.

#### 3.2.2. Variable Definitions

COVID-19 (*COVID*) and the top two livestock companies (*BIG*) are the dummy variables of this study’s livestock industry revenue model. This study uses the revenue to define large livestock companies. From 2015 to 2020, the revenue of China’s top two large livestock enterprises accounted for a relatively high proportion of the industry’s revenue. This study takes the top two livestock companies (*BIG*) as one of the dummy variables of the model. Under the pandemic, this study can use *BIG* to explore the impact of *COVID* on large and non-large livestock companies. We bring together the definitions of the above variables and other variables in Table 1.

### 3.3. Estimation Model

The livestock industry estimation model of this study is as follows:

(6) $$\begin{array}{cc} lnREVENUE= & \alpha_{0}+\alpha_{1}lnMSTAFF+\alpha_{2}lnRSTAFF+\alpha_{3}lnOSTAFF+\beta_{1}lnFIXED+\delta_{1}lnDEVELOP\\ & +\epsilon_{1}lnBIOLOGY+\frac{1}{2}\;\alpha_{11}\;{\left(ln\;MSTAFF\right)}^{2}+\frac{1}{2}\;\alpha_{22}\;{\left(ln\;RSTAFF\right)}^{2}+\frac{1}{2}\;\alpha_{33}\;{\left(ln\;OSTAFF\right)}^{2}\\ & +\frac{1}{2}\;\beta_{11}\;{\left(ln\;FIXED\right)}^{2}+\frac{1}{2}\;\delta_{11}\;{\left(ln\;DEVELOP\right)}^{2}+\frac{1}{2}\;\epsilon_{11}\;{\left(ln\;BIOLOGY\right)}^{2}\\ & +\alpha_{12}lnMSTAFFlnRSTAFF+\alpha_{13}lnMSTAFFlnOSTAFF+\alpha_{23}\;ln\;RSTAFF\;ln\;OSTAFF\\ & +\gamma_{11}lnMSTAFFlnFIXED+\gamma_{21}\;ln\;RSTAFF\;ln\;FIXED+\gamma_{31}\;ln\;OSTAFF\;ln\;FIXED\\ & +\varepsilon_{11}lnMSTAFFlnDEVELOP+\varepsilon_{21}ln\;RSTAFF\;ln\;DEVELOP+\varepsilon_{31}\;ln\;OSTAFF\;ln\;DEVELOP\\ & +\mu_{11}lnMSTAFFlnBIOLOGY+\mu_{21}lnRSTAFFlnBIOLOGY+\mu_{31}\;ln\;OSTAFFlnBIOLOGY\\ & +\theta_{11}\;ln\;FIXED\;ln\;DEVELOP+\rho_{11}\;ln\;FIXED\;ln\;BIOLOGY+\sigma_{11}\;ln\;DEVELOP\;ln\;BIOLOGY\\ & +\varphi_{1}\;BIG+\varphi_{2}\;COVID+\varphi_{3}\;BIG\;COVID \end{array}$$

The following is the APE derivation process of variables:

The APE of *MSTAFF* on *REVENUE*:

(7) $$\begin{array}{cc} \partial \hat{\;ln\;REVENUE}/ & \partial \;ln\;MSTAFF\\ & ={\hat{\alpha }}_{1}+{\hat{\alpha }}_{11}\;\bar{ln\;MSTAFF}+{\hat{\alpha }}_{12}\;\bar{ln\;RSTAFF}\\ & +{\hat{\alpha }}_{13}\;\bar{ln\;OSTAFF}+{\hat{\gamma }}_{11}\;\bar{ln\;FIXED}\\ & +{\hat{\varepsilon }}_{11}\;\bar{ln\;DEVELOP}+{\hat{\mu }}_{11}\;\bar{ln\;BIOLOGY}\; \end{array}$$

The APE of *RSTAFF* on *REVENUE:*

(8) $$\begin{array}{cc} \partial \hat{\;ln\;REVENUE}/ & \partial \;ln\;RSTAFF\\ & ={\hat{\alpha }}_{2}+{\hat{\alpha }}_{22}\;\bar{ln\;RSTAFF}+{\hat{\alpha }}_{12}\;\bar{ln\;MSTAFF}\\ & +{\hat{\alpha }}_{23}\;\bar{lnOSTAFF}+{\hat{\alpha }}_{21}\;\bar{ln\;FIXED}\\ & +{\hat{\varepsilon }}_{21}\;\bar{ln\;DEVELOP}+{\hat{\mu }}_{21}\;\bar{ln\;BIOLOGY} \end{array}$$

The APE of *OSTAFF* on *REVENUE:*

(9) $$\begin{array}{cc} \partial \hat{\;ln\;REVENUE}/ & \partial \;ln\;OSTAFF\\ & ={\hat{\alpha }}_{3}+{\hat{\alpha }}_{33}\;\bar{ln\;OSTAFF}+{\hat{\alpha }}_{13}\;\bar{ln\;MSTAFF}\\ & +{\hat{\alpha }}_{23}\;\bar{ln\;RSTAFF}+{\hat{\gamma }}_{31}\;\bar{ln\;FIXED}\\ & +{\hat{\varepsilon }}_{31}\;\bar{ln\;DEVELOP}+{\hat{\mu }}_{31}\;\bar{ln\;BIOLOGY} \end{array}$$

The APE of *FIXED* on *REVENUE:*

(10) $$\begin{array}{cc} \partial \hat{\;ln\;REVENUE}/ & \partial \;ln\;FIXED\\ & ={\hat{\beta }}_{1}+{\hat{\beta }}_{11}\;\bar{ln\;FIXED}+{\hat{\gamma }}_{11}\;\bar{ln\;MSTAFF}\\ & +{\hat{\gamma }}_{21}\;\bar{ln\;RSTAFF}+{\hat{\gamma }}_{31}\;\bar{ln\;OSTAFF}\\ & +{\hat{\theta }}_{11}\bar{ln\;DEVELOP}+{\hat{\rho }}_{11}\;\bar{ln\;BIOLOGY} \end{array}$$

The APE of *DEVELOP* on *REVENUE:*

(11) $$\begin{array}{cc} \partial \hat{\;ln\;REVENUE}/ & \partial \;ln\;DEVELOP\\ & ={\hat{\delta }}_{1}+{\hat{\delta }}_{11}\;\bar{ln\;DEVELOP}\\ & +{\hat{\varepsilon }}_{11}\;\bar{ln\;MSTAFF}+{\hat{\varepsilon }}_{21}\;\bar{ln\;RSTAFF}\\ & +{\hat{\varepsilon }}_{31}\;\bar{ln\;OSTAFF}+{\hat{\theta }}_{11}\;\bar{ln\;FIXED}\\ & +{\hat{\sigma }}_{11}\;\bar{ln\;BIOLOGY} \end{array}$$

The APE of *BIOLOGY* on *REVENUE:*

(12) $$\begin{array}{cc} \partial \hat{\;ln\;REVENUE}/ & \partial \;ln\;BIOLOGY\\ & ={\hat{\epsilon }}_{1}+{\hat{\epsilon }}_{11}\;\bar{ln\;BIOLOGY}\\ & +{\hat{\mu }}_{11}\;\bar{ln\;MSTAFF}+{\hat{\mu }}_{21}\;\bar{ln\;RSTAFF}\\ & +{\hat{\mu }}_{31}\;\bar{ln\;OSTAFF}+{\hat{\rho }}_{11}\;\bar{ln\;FIXED}\\ & +{\hat{\sigma }}_{11}\;\bar{ln\;DEVELOP} \end{array}$$

The APE of *BIG* on *REVENUE:*

(13) $$\partial \hat{\;ln\;REVENUE}/\partial \;BIG=\varphi_{1}+\varphi_{3}\;COVID$$

The APE of *COVID* on *REVENUE:*

(14) $$\partial \hat{\;ln\;REVENUE}/\partial \;COVID=\varphi_{2}+\varphi_{3}\;BIG$$

## 4. Results

### 4.1. Descriptive Statistics and Correlation Matrix

Table 2 shows the descriptive statistics. It can be seen that during the study period, the medians of revenue, R&D personnel, ordinary personnel, net fixed assets, R&D investment, and net productive biological assets are all less than the average. From the perspective of the average revenue of the livestock industry every year, the revenue of livestock products in 2015–2019 shows an upward trend year by year, showing the increase in consumer demand for livestock products. However, under the impact of the 2020 pandemic, the consumption of livestock products has declined, and the average revenue of Chinese livestock companies has also declined significantly. In 2020, livestock companies continue to expand investment in fixed assets, productive biological assets, and human resources. This shows that China’s livestock industry generally believes that the decline in livestock products sales caused by the COVID-19 pandemic is temporary.

Table 3 shows the Spearman and Pearson correlation coefficients (On the right is Pearson, and on the left is Spearman). The values in Table 3 indicate a positive correlation between *COVID* and *REVENUE,* but the correlation is drawn without controlling for other variables. After we control other variables, we can analyze the impact of *COVID* on *REVENUE* through APE, and we will have a more accurate conclusion. In addition, *COVID* is significantly positively correlated with *OSTAFF*.

### 4.2. The Pandemic and the Revenue of Livestock Industry

This study analyzes the impact of COVID-19 on the revenue of the livestock industry. Taking the time of COVID-19 occurrence in 2020 as the boundary, the Chow Test is used to explore the relationship between the pandemic and the revenue of the livestock industry, the F statistics were 2.16. The rejection of the null hypothesis indicates that the COVID-19 would impact the revenue of the livestock industry. Meanwhile, this study puts *BIG* as a dummy variable into the model.

### 4.3. Estimation Results

#### 4.3.1. The Revenue Function

Table 4 shows the estimated results of Chinese livestock enterprises’ revenue functions. This study uses the more flexible translog format instead of using the simple Cobb-Douglas format (Log-linear). We needed to check whether the translog format can provide a correct representation of Chinese livestock enterprises’ revenue function. Therefore, this study tests whether these conditions in Equation (6) is met:

(15) $$\alpha_{il}=\beta_{11}=\delta_{11}=\epsilon_{11}=\gamma_{i1}=\varepsilon_{i1}=\mu_{i1}=\theta_{11}=\rho_{11}=\sigma_{11}=0\;\mathrm{for}\;\mathrm{all}\;i=1,2,3.$$

Table 4 shows the result of the F statistical value is 3.71, which significantly rejects the null hypothesis of the log-linear specification, indicating that the translog format is suitable for analyzing Chinese livestock enterprises’ revenue functions.

#### 4.3.2. Livestock Product Sales

Table 5 shows the average partial effect (APE). The APE of *COVID* to the *REVENUE* of Chinese livestock enterprises is negative and significant. Meanwhile, Table 4 shows that the estimated value of the *COVID* coefficient is significantly negative in the revenue function equation of Chinese livestock enterprises. These figures show that the COVID-19 pandemic reduces livestock product sales.

We analyzed the possible reason is that the inconvenience of transportation caused by the pandemic makes people spend less time shopping to buy livestock products. Another reason that cannot be ignored is the decrease in income brought about by the pandemic, which has caused consumers to reduce their consumption of livestock products and instead save their income.

The APE of *COVID* on *BIG* and non-*BIG* are −0.254 and −0.387, respectively. These results indicate that under the pandemic, the decline in revenue of large livestock enterprises is lower than that of small- and medium-sized livestock enterprises. Hypothesis 1 of this study has been verified by the above contents. In China, large-scale livestock companies have further improved their Internet marketing channels during the pandemic, and the livestock products produced by them are better packaged for preservation. Therefore, in the predicament of the pandemic, although the overall consumption of livestock products has dropped significantly, the consumption of livestock products produced by large enterprises has decreased by a smaller amount. This result may provide inspiration for managers of small and medium livestock companies. It is possible that small- and medium-sized livestock companies can significantly reduce the reduction in livestock product sales caused by the pandemic by establishing a comprehensive door-to-door service and online sales system similar to large-scale livestock companies.

## 5. Conclusions

Due to the decline in China’s livestock production during the pandemic, China has increased its imports of foreign livestock products. According to data released by the General Administration of Customs of China, in 2020, China’s total meat imports totaled 9.91 million tons, an increase of 60.4% year on year. Among them, pork imports were 4,392,200 tons, a year-on-year increase of 108.34%; chicken imports were 1,433,000 tons, a year-on-year increase of 98.28%; beef imports were 2,118,300 tons, a year-on-year increase of 27.65%. According to Euromonitor data, the market size of China’s imported meat products in 2020 is 15 billion US dollars, and Brazil is China’s largest source of meat imports, accounting for up to 21%. Brazil’s meat exports to China have proliferated, from USD 800 million in 2015 to USD 3.1 billion in 2020, with an average annual growth rate of 31%. During the pandemic, the consumption demand for meat products in Brazil’s domestic market decreased, and prices were far below the international market level. At the same time, during the pandemic, the output of Chinese livestock products has dropped significantly. The Chinese market can form a benign interaction with the export of Brazilian livestock companies.

Due to human mobility restrictions and rising unemployment, the COVID-19 crisis has impacted livestock production, negatively impacting many livestock companies and farmers. We need to take measures to promote the livestock industry’s development better. The following options are available for policymakers and competent authorities to consider: (1) in terms of transportation, providing animal product transport drivers with special transportation permits, and promoting the easier import and export of animal products under the premise of quarantine safety; (2) under the premise of ensuring epidemic prevention requirements, allowing more markets and supermarkets (especially fresh markets and fresh supermarkets) to resume opening; (3) improving the level of aseptic packaging and refrigerated transportation of animal products; (4) formulating policies that require livestock workers to abide by certain epidemic prevention codes of conduct [55]; (5) providing children with high-quality livestock products at low prices can increase the revenue of livestock enterprises and farmers and further protect the health and nutritional level of children under the pandemic; (6) Support livestock enterprises through special subsidies from dedicated financial institutions; (7) helping producers improve online sales channels to promote the sales of animal products and the growth of enterprise revenue.

This study is limited to analysis from the perspective of the financial statements of listed companies in China’s livestock industry. Future study may consider conducting cross-regional comparative studies on the livestock industry in different countries, which may obtain the different impacts of COVID-19 on the livestock industry in different economies.

## Figures and Tables

**Table 1:** Variable definitions.

Variable		Definition
Theoretical Variable	Proxy Variable	
*r*	*REVENUE*	Total revenue of enterprises
*x*_1_	*MSTAFF*	Total number of management personnel
*x*_2_	*RSTAFF*	Total number of research and development personnel
*x*_3_	*OSTAFF*	Total number of ordinary personnel
	*EMPLOYEE*	Total number of employees
*f*	*FIXED*	Net fixed assets
*d*	*DEVELOP*	R&D investment
*b*	*BIOLOGY*	Net productive biological assets
	*BIG*	A dummy variable that equals one if the livestock enterprise is one of China’s top two livestock enterprises, and 0 otherwise
	*COVID*	A dummy variable. In 2020, *COVID* is 1; in 2015-2019, *COVID* is 0

**Table 2:** Descriptive statistics.

Panel A:	2015 (*n* = 41)					2016 (*n* = 44)				
Variables	Mean	Median	Max	Min	Std. Dev.	Mean	Median	Max	Min	Std. Dev.
*REVENUE*	¥3310.00	¥901.00	¥48,200.00	¥149.00	¥7950.00	¥5890.00	¥1510.00	¥59,400.00	¥141.00	¥11,600.00
*MSTAFF*	14.73	15.00	20.00	8.00	3.15	14.55	14.00	20.00	8.00	3.32
*RSTAFF*	288.32	121.00	1265.00	14.00	406.06	379.27	128.00	1349.00	22.00	480.48
*OSTAFF*	6171.07	2723.00	42,754.00	1116.00	8542.89	10,344.55	2834.00	47,945.00	1035.00	13,876.26
*EMPLOYEE*	6474.12	2787.00	44,039.00	1228.00	8701.07	10,738.36	2937.00	49,314.00	1189.00	14,182.35
*FIXED*	¥2110.00	¥937.00	¥9370.00	¥517.00	¥2250.00	¥3110.00	¥1360.00	¥11,400.00	¥584.00	¥3240.00
*DEVELOP*	¥39.78	¥22.94	¥194.00	¥2.04	¥59.29	¥58.35	¥22.33	¥208.00	¥3.39	¥70.46
*BIOLOGY*	¥208.00	¥200.00	¥2540.00	¥0.18	¥391.00	¥460.00	¥194.00	¥3090.00	¥0.18	¥792.00
Panel B:	2017 (*n* = 44)					2018 (*n* = 43)				
Variables	Mean	Median	Max	Min	Std. Dev.	Mean	Median	Max	Min	Std. Dev.
*REVENUE*	¥5970.00	¥1530.00	¥55,700.00	¥154.00	¥10,800.00	¥6820.00	¥2060.00	¥57,200.00	¥147.00	¥11,300.00
*MSTAFF*	15.00	15.00	22.00	8.00	3.45	14.49	14.00	23.00	8.00	3.62
*RSTAFF*	467.82	166.00	1524.00	19.00	542.95	486.70	210.00	1575.00	19.00	553.51
*OSTAFF*	11,650.09	3179.00	49,028.00	840.00	14,876.02	12,342.67	4173.00	47,041.00	494.00	14,547.71
*EMPLOYEE*	12,132.91	3552.00	50,574.00	1075.00	15,249.31	12,843.86	4397.00	48,639.00	658.00	14,932.17
*FIXED*	¥3940.00	¥1440.00	¥14,400.00	¥559.00	¥4090.00	¥5240.00	¥2170.00	¥18,300.00	¥536.00	¥5220.00
*DEVELOP*	¥82.25	¥62.56	¥307.00	¥2.18	¥96.14	¥105.00	¥49.55	¥553.00	¥1.96	¥157.00
*BIOLOGY*	¥617.00	¥248.00	¥3300.00	¥0.31	¥925.00	¥740.00	¥279.00	¥3620.00	¥0.23	¥1030.00
Panel C:	2019 (*n* = 40)					2020 (*n* = 43)				
Variables	Mean	Median	Max	Min	Std. Dev.	Mean	Median	Max	Min	Std. Dev.
*REVENUE*	¥8980.00	¥3430.00	¥73,100.00	¥214.00	¥14,200.00	¥7830.00	¥1890.00	¥56,300.00	¥427.00	¥13,000.00
*MSTAFF*	13.70	13.50	22.00	8.00	3.45	13.65	14.00	18.00	8.00	2.80
*RSTAFF*	491.30	167.50	1620.00	23.00	594.30	654.70	134.00	3136.00	8.00	1045.80
*OSTAFF*	16,359.80	4791.00	49,520.00	1450.00	18,054.09	21,197.14	5623.00	119,615.00	1303.00	34,678.70
*EMPLOYEE*	16,864.80	4973.50	50,319.00	1487.00	18,536.36	21,865.49	5772.00	121,995.00	1440.00	35,530.86
*FIXED*	¥6460.00	¥2500.00	¥22,700.00	¥525.00	¥6740.00	¥6830.00	¥2550.00	¥58,500.00	¥498.00	¥11,500.00
*DEVELOP*	¥135.00	¥65.59	¥570.00	¥5.32	¥184.00	¥122.00	¥27.34	¥528.00	¥4.57	¥175.00
*BIOLOGY*	¥997.00	¥320.00	¥5100.00	¥0.42	¥1410.00	¥1590.00	¥280.00	¥9480.00	¥0.53	¥2670.00

Note: Currency unit: millions of RMB. ¥—renminbi (currency of the Republic of China).

**Table 3:** Correlation statistics.

	(1)	(2)	(3)	(4)	(5)	(6)	(7)	(8)	(9)	(10)
(1) *REVENUE*	1.000	0.280	0.691	0.705	0.710	0.727	0.734	0.752	0.698	0.053
	-----	(0.000)	(0.000)	(0.000)	(0.000)	(0.000)	(0.000)	(0.000)	(0.000)	(0.398)
(2) *MSTAFF*	0.049	1.000	0.112	0.231	0.229	0.218	0.084	0.118	−0.037	−0.097
	(0.433)	-----	(0.074)	(0.000)	(0.000)	(0.000)	(0.182)	(0.059)	(0.553)	(0.124)
(3) *RSTAFF*	0.691	0.162	1.000	0.744	0.758	0.689	0.871	0.839	0.773	0.135
	(0.000)	(0.010)	-----	(0.000)	(0.000)	(0.000)	(0.000)	(0.000)	(0.000)	(0.031)
(4) *OSTAFF*	0.786	−0.075	0.568	1.000	1.000	0.933	0.717	0.817	0.639	0.188
	(0.000)	(0.235)	(0.000)	-----	(0.000)	(0.000)	(0.000)	(0.000)	(0.000)	(0.003)
(5) *EMPLOYEE*	0.807	−0.028	0.627	0.993	1.000	0.933	0.727	0.824	0.648	0.187
	(0.000)	(0.655)	(0.000)	(0.000)	-----	(0.000)	(0.000)	(0.000)	(0.000)	(0.003)
(6) *FIXED*	0.783	−0.068	0.736	0.869	0.885	1.000	0.693	0.764	0.568	0.155
	(0.000)	(0.281)	(0.000)	(0.000)	(0.000)	-----	(0.000)	(0.000)	(0.000)	(0.013)
(7) *DEVELOP*	0.736	0.052	0.837	0.680	0.708	0.685	1.000	0.812	0.823	0.107
	(0.000)	(0.404)	(0.000)	(0.000)	(0.000)	(0.000)	-----	(0.000)	(0.000)	(0.088)
(8) *BIOLOGY*	0.730	0.065	0.753	0.749	0.769	0.750	0.861	1.000	0.644	0.255
	(0.000)	(0.303)	(0.000)	(0.000)	(0.000)	(0.000)	(0.000)	-----	(0.000)	(0.000)
(9) *BIG*	0.613	−0.037	0.612	0.565	0.571	0.562	0.642	0.547	1.000	−0.003
	(0.000)	(0.552)	(0.000)	(0.000)	(0.000)	(0.000)	(0.000)	(0.000)	-----	(0.968)
(10) *COVID*	0.095	−0.088	0.001	0.167	0.163	0.064	0.061	0.084	-0.003	1.000
	(0.128)	(0.163)	(0.984)	(0.007)	(0.009)	(0.311)	(0.329)	(0.184)	(0.968)	-----

Note: (1) is *REVENUE*; (2) is *MSTAFF*; (3) is *RSTAFF*; (4) is *OSTAFF*; (5) is *EMPLOYEE*; (6) is *FIXED*; (7) is *DEVELOP*; (8) is *BIOLOGY*; (9) is *BIG*; (10) is *COVID*.

**Table 4:** Translog estimates.

$\begin{array}{cc} lnREVENUE=\alpha_{0} & +\alpha_{1}lnMSTAFF+\alpha_{2}lnRSTAFF+\alpha_{3}lnOSTAFF+\beta_{1}lnFIXED+\delta_{1}lnDEVELOP+\epsilon_{1}lnBIOLOGY\\ & +\frac{1}{2}\;\alpha_{11}\;{\left(ln\;MSTAFF\right)}^{2}+\frac{1}{2}\;\alpha_{22}\;{\left(ln\;RSTAFF\right)}^{2}+\frac{1}{2}\;\alpha_{33}\;{\left(ln\;OSTAFF\right)}^{2}+\frac{1}{2}\;\beta_{11}\;{\left(ln\;FIXED\right)}^{2}+\frac{1}{2}\;\delta_{11}\;{\left(ln\;DEVELOP\right)}^{2}\\ & +\frac{1}{2}\;\epsilon_{11}\;{\left(ln\;BIOLOGY\right)}^{2}+\alpha_{12}lnMSTAFFlnRSTAFF+\alpha_{13}lnMSTAFFlnOSTAFF+\alpha_{23}\;ln\;RSTAFF\;ln\;OSTAFF\\ & +\gamma_{11}lnMSTAFFlnFIXED+\gamma_{21}\;ln\;RSTAFF\;ln\;FIXED+\gamma_{31}\;ln\;OSTAFF\;ln\;FIXED\\ & +\varepsilon_{11}lnMSTAFFlnDEVELOP+\varepsilon_{21}ln\;RSTAFF\;ln\;DEVELOP+\varepsilon_{31}\;ln\;OSTAFF\;ln\;DEVELOP\\ & +\mu_{11}lnMSTAFFlnBIOLOGY+\mu_{21}lnRSTAFFlnBIOLOGY+\mu_{31}\;ln\;OSTAFFlnBIOLOGY\\ & +\theta_{11}\;ln\;FIXED\;ln\;DEVELOP+\rho_{11}\;ln\;FIXED\;ln\;BIOLOGY+\sigma_{11}\;ln\;DEVELOP\;ln\;BIOLOGY+\varphi_{1}\;BIG+\varphi_{2}\;COVID\\ & +\varphi_{3}\;BIG\;COVID \end{array}$			
**Variables**	**Coefficient**	**Variables**	**Coefficient**
	***t*-Statistic**		***t*-Statistic**
Intercept	269.480	(ln*MSTAFF*)(ln*DEVELOP*)	−1.920 ***
	(2.224)		(−3.532)
ln*MSTAFF*	1.860	(ln*MSTAFF*)(ln*BIOLOGY*)	−0.376
	(0.103)		(−0.782)
ln*RSTAFF*	4.321	(ln*RSTAFF*)(ln*OSTAFF*)	1.013 ***
	(0.711)		(3.280)
ln*OSTAFF*	24.538 **	(ln*RSTAFF*)(ln*FIXED*)	−0.950 ***
	(2.404)		(−2.734)
ln*FIXED*	−35.264 **	(ln*RSTAFF*)(ln*DEVELOP*)	0.102
	(−2.553)		(0.693)
ln*DEVELOP*	−0.301	(ln*RSTAFF*)(ln*BIOLOGY*)	0.228 **
	(−0.061)		(2.123)
ln*BIOLOGY*	0.375	(ln*OSTAFF*)(ln*FIXED*)	−0.642
	(0.120)		(−1.242)
(ln*MSTAFF*) ^2^	1.979	(ln*OSTAFF*)(ln*DEVELOP*)	−0.873 ***
	(1.113)		(−2.945)
(ln*RSTAFF*) ^2^	−0.079	(ln*OSTAFF*)(ln*BIOLOGY*)	−0.055
	(−0.714)		(−0.663)
(ln*OSTAFF*) ^2^	−0.174	(ln*FIXED*)(ln*DEVELOP*)	0.809 ***
	(−0.880)		(2.660)
(ln*FIXED*) ^2^	0.659 *	(ln*FIXED*)(ln*BIOLOGY*)	0.112
	(1.710)		(0.690)
(ln*DEVELOP*) ^2^	−0.098	(ln*DEVELOP*)(ln*BIOLOGY*)	−0.072
	(−1.059)		(−1.032)
(ln*BIOLOGY*) ^2^	−0.032	*BIG*	0.440
	(−1.136)		(1.218)
(ln*MSTAFF*)(ln*RSTAFF*)	0.796	*COVID*	−0.387 ***
	(1.124)		(−2.937)
(ln*MSTAFF*)(ln*OSTAFF*)	1.081	*BIGCOVID*	0.133
	(1.534)		(0.417)
(ln*MSTAFF*)(ln*FIXED*)	0.745		
	(0.929)		
Adjusted R–squared	0.799		
Degrees of freedom	255		
*The null hypothesis * (*log-linear*) ($\alpha_{il}=\beta_{11}=\delta_{11}=\epsilon_{11}=\gamma_{i1}=\varepsilon_{i1}=\mu_{i1}=\theta_{11}=\rho_{11}=\sigma_{11}=0$)			
*F*–statistic value	3.71		
Significance level	0.000		

Note: 1. ***, **, * Denotes significantly difference from zero at the 1%, 5%, and 10% levels, respectively. ^2^. All variables’ definitions are the same as Table 1. ^2^—represents the meaning of square.

**Table 5:** APE of variables on *REVENUE*.

APE	Value	Significance Test
*APE_MSTAFF*	1.794	$H_{0}:\alpha_{1}=\alpha_{11}=\alpha_{12}=\alpha_{13}=\gamma_{11}=\varepsilon_{11}=\mu_{11}=0$
		*F*-statistic value = 3.49
		Significance level = 0.00
*APE_RSTAFF*	−0.035	$H_{0}:\alpha_{2}=\alpha_{22}=\alpha_{12}=\alpha_{23}=\gamma_{21}=\varepsilon_{21}=\mu_{21}=0$
		*F*-statistic value = 3.50
		Significance level = 0.00
*APE_OSTAFF*	−0.348	$H_{0}:\alpha_{3}=\alpha_{33}=\alpha_{13}=\alpha_{23}=\gamma_{31}=\varepsilon_{31}=\mu_{31}=0$
		*F*-statistic value = 7.48
		Significance level = 0.00
*APE_FIXED*	0.910	$H_{0}:\beta_{1}=\beta_{11}=\gamma_{11}=\gamma_{21}=\gamma_{31}=\theta_{11}=\rho_{11}=0$
		*F*-statistic value = 5.58
		Significance level = 0.00
*APE_DEVELOP*	0.196	$H_{0}:\delta_{1}=\delta_{11}=\varepsilon_{11}=\varepsilon_{21}=\varepsilon_{31}=\theta_{11}=\sigma_{11}=0$
		*F*-statistic value = 4.06
		Significance level = 0.00
*APE_BIOLOGY*	0.024	$H_{0}:\epsilon_{1}=\epsilon_{11}=\mu_{11}=\mu_{21}=\mu_{31}=\rho_{11}=\sigma_{11}=0$
		*F*-statistic value = 2.12
		Significance level = 0.04
*APE_BIG*		$H_{0}:\varphi_{1}=\varphi_{3}=0$
When *COVID* = 0	0.440	*F*-statistic value = 0.96
When *COVID* = 1	0.572	Significance level = 0.38
*APE_COVID*		$H_{0}:\varphi_{2}=\varphi_{3}=0$
When *BIG* = 0	−0.387	*F*-statistic value = 4.79
When *BIG* = 1	−0.254	Significance level = 0.01

## Data Availability

All relevant data are within the manuscript.
